# Editorial: Sports policy and management in the era of sustainable development goals: challenges and opportunities

**DOI:** 10.3389/fspor.2026.1862483

**Published:** 2026-05-25

**Authors:** David Cabello-Manrique, Athanasios (Sakis) Pappous, Jordi Segui-Urbaneja, Juan Carlos Guevara-Pérez

**Affiliations:** 1 Department of Physical Education and Sports, Faculty of Sport Science, iMUDS, University of Granada, Granada, Spain; 2 Universita di Bologna, Bologna, Italy; 3 Sport Management Department, Institut Nacional Educació Física de Catalunya, Lleida, Spain; 4 Department of Accounting and Finance, University of Zaragoza, Zaragoza, Spain

**Keywords:** accountability, governance, public policy, social change, sport for development (SfD), sport management, sustainable development goals

The adoption of the United Nations 2030 Agenda has contributed to repositioning sport within broader debates on public policy, governance, and social transformation. Sport is no longer understood solely as a domain of competition, performance, or leisure; it is increasingly framed as a field with the potential to contribute to health promotion, social inclusion, gender equality, environmental sustainability, institutional quality, and community development. At the same time, this expanded role brings renewed responsibilities for policymakers, managers, and researchers. If sport is to contribute meaningfully to sustainable development, it must be supported by sound governance, coherent policy design, effective implementation, and appropriate mechanisms for evaluation.

This Research Topic was conceived precisely in response to that challenge. It brings together contributions that examine sport policy and management from multiple angles, geographical contexts, and methodological traditions, while maintaining a shared concern: how sport can respond to the opportunities and tensions created by the Sustainable Development Goals (SDGs). Collectively, the articles show that sport's developmental value should never be taken for granted. Its contribution depends on institutional arrangements, regulatory frameworks, management practices, resource allocation, and the capacity to connect sport systems with wider societal objectives.

A first line of inquiry running through this Research Topic concerns governance, accountability, and regulation in sport organizations and systems. These issues are central to the SDG era, as expectations of transparency, responsibility, and long-term sustainability increasingly shape sport governance at all levels. Contributions in this Topic show that governance is not a secondary or technical matter, but a decisive condition for whether sport organizations can act in ways that are socially legitimate and strategically sustainable. For example, *Interlinking financial stability regulation and governance in German professional soccer: contribution and implications* highlights the governance value of financial discipline, while also pointing to the need for stronger harmonization and more effective incentive structures. Similarly, *Tennis club governance: an international comparison of Southern European clubs* reveals substantial differences in democracy, participation, transparency, and oversight, illustrating that governance quality remains uneven even in established sport settings. In a different but equally relevant area, *Governance of prepaid consumption in China's sports industry based on collaborative governance theory: theoretical framework, realistic dilemmas, and practical pathways* argues for collaborative governance, stronger legal protections, improved regulatory tools, and more robust mechanisms for safeguarding consumers. Taken together, these studies reinforce a common message: sustainable development in sport requires governance frameworks that are not only formally established, but also credible, inclusive, and operationally effective.

A second major theme relates to public policy design and the role of sport within broader development strategies. Several contributions demonstrate that sport policy must be understood as part of larger systems of welfare, public health, territorial development, and social equity. *Study on the impact of rural public sports facilities and instructors on residents' participation in sports activities in China* shows how participation in sport is shaped not only by individual motivation, but also by the presence of infrastructure, institutional support, and human guidance, especially in areas affected by structural disadvantage. Complementing this perspective, *Policy tools, objectives, and governance in China's public sports services: an X–Y–Z framework analysis of national and provincial policies* identifies imbalances in the use of policy tools and objectives, showing that supply-oriented approaches remain dominant while responsiveness, equity, and innovation receive less sustained attention. On the other side of the planet, *Physical activity, sports participation, and sustainable development in the Ibero-American region: a pilot implementation of indicators in Chile, Costa Rica, and Ecuador* offers an especially valuable contribution by moving beyond rhetorical commitments and toward measurable frameworks for assessing the contribution of sport and physical activity to national development. Together, these articles remind us that the relationship between sport and the SDGs must be built through policy capacity, administrative coordination, and better data systems. Ambitious discourse alone is not enough; sustainable development also depends on the ability to monitor, compare, and improve public action over time.

A third thematic block in this Research Topic focuses on sport for development, inclusion, and social change. Here, the published articles engage with a long-standing yet still contested question: under what conditions can sport contribute to broader forms of human and community development? *Ripples of change: transforming lives and communities through sport in Colombia* offers rich evidence of sport-based interventions generating positive effects on life skills, social cohesion, non-violent communication, and civic values, with benefits extending beyond participants to families and communities. *Playing for progress: policy advocacy in sport for development* broadens this discussion by showing that many organizations do engage with advocacy, but often in ways centered on funding security and organizational survival. Its call for more progressive and structurally oriented advocacy is particularly significant, because it challenges the field to move beyond micro-level interventions and to engage more directly with the policy environments that shape inequality and opportunity. “*It should just be about sport!”: exploring Italian athletes' perspectives in paralympic media coverage* adds another essential dimension by emphasizing representation. It shows that improved visibility does not necessarily eliminate stereotypical narratives and that more inclusive, athlete-centered communication remains a key challenge. In this sense, sustainable development through sport must also include cultural recognition, voice, and fair representation.

A fourth and highly relevant theme involves sustainability, strategic development, and the contradictions of growth in contemporary sport systems. The SDGs have encouraged sport organizations and policymakers to place greater emphasis on environmental responsibility and long-term social value, yet the path toward sustainability is often uneven and contested. *Green practices and sustainability as precursors to the intention to participate again in sporting events held in nature* shows that green practices and sustainability are not peripheral concerns, but factors that influence participants' perceptions of quality, satisfaction, and intention to return. At the same time, other contributions reveal that expansion and visibility do not automatically translate into sustainable progress. *Involution and governance reform in professional football club–school integration: a case study of S City's youth training base* identifies a pattern of quantitative growth without corresponding qualitative development, illustrating how partnership expansion can coexist with weaknesses in talent formation and governance depth. Likewise, *The take-off of the Saudi professional football league in the context of the 2030 vision: effect on the competitive balance* suggests that rapid internationalization and capital concentration may enhance visibility while simultaneously weakening competitive balance. This line of reflection is extended by *Thinking out of the (Olympic) box: is It time to reconsider permanent hosting for sustainable Olympics? A conceptual review*, which argues that the sustainability crisis of the Olympic Games should be understood as a structural “wicked problem,” one that cannot be solved through incremental reform alone. By questioning the long-standing rotational hosting model and exploring permanent or semi-permanent hosting as a transformative alternative, this contribution invites scholars and policymakers to reconsider how mega-event governance, legacy, environmental impact, and global equity might be more sustainably aligned. Taken together, these studies highlight one of the core tensions of the SDG era in sport: the need to reconcile growth with equity, ambition with accountability, and international projection with durable institutional foundations.

Overall, the articles gathered in this Research Topic confirm that sport policy and management research is entering a phase of greater maturity and wider societal relevance. Rather than approaching sport as an inherently positive force, the contributions in this collection treat it as a field whose effects are shaped by governance choices, policy priorities, institutional capacities, and cultural narratives. This shift is crucial. The SDG framework invites not only optimism about what sport might contribute, but also critical reflection on the conditions under which those contributions become possible, equitable, and sustainable.

For that reason, this collection offers more than a set of isolated case studies. It provides a broader picture of a field in transformation, one in which questions of regulation, participation, legitimacy, inclusion, measurement, and sustainability increasingly intersect. As Topic Editors, we hope that the articles published here will stimulate further international and interdisciplinary dialogue, encourage more evidence-informed policy and management practices, and contribute to a deeper understanding of how sport can respond to the urgent social, institutional, and environmental challenges of our time.

